# {2,2′-[Cyclo­hexane-1,2-diylbis(nitrilo­methyl­idyne)]diphenolato}nickel(II)

**DOI:** 10.1107/S1600536809006084

**Published:** 2009-02-25

**Authors:** Chunbao Tang

**Affiliations:** a Department of Chemistry, Jiaying University, Meizhou 514015, People’s Republic of China

## Abstract

In the title mononuclear nickel(II) complex, [Ni(C_20_H_20_N_2_O_2_)], the Ni atom is four-coordinated in a square-planar geometry by the four donor atoms of the Schiff base ligand. The dihedral angle between the two benzene rings is 9.4 (2)°. The cyclo­hexyl group adopts a *C*-form chair conformation.

## Related literature

For nickel(II) complexes in bio-inorganic chemistry and coordination chemistry, see: Angulo *et al.* (2001 ▶); Dey *et al.* (2004 ▶); Edison *et al.* (2004 ▶); Ramadevi *et al.* (2005 ▶); Suh *et al.* (1996 ▶). For puckering parameters, see: Cremer & Pople (1975 ▶).
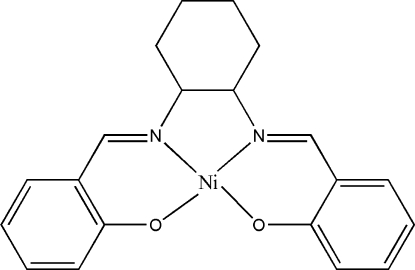


## Experimental

### 

#### Crystal data

[Ni(C_20_H_20_N_2_O_2_)]
*M* *_r_* = 379.09
Monoclinic, 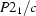

*a* = 7.6193 (8) Å
*b* = 19.118 (2) Å
*c* = 11.5459 (12) Å
β = 90.907 (1)°
*V* = 1681.6 (3) Å^3^
*Z* = 4
Mo *K*α radiation
μ = 1.17 mm^−1^
*T* = 298 K
0.30 × 0.30 × 0.28 mm

#### Data collection

Bruker SMART CCD area-detector diffractometer
Absorption correction: multi-scan (*SADABS*; Sheldrick, 1996 ▶) *T*_min_ = 0.721, *T*_max_ = 0.735
9694 measured reflections
3650 independent reflections
3023 reflections with *I* > 2σ(*I*)
*R*_int_ = 0.022

#### Refinement

*R*[*F* ^2^ > 2σ(*F* ^2^)] = 0.027
*wR*(*F* ^2^) = 0.069
*S* = 1.05
3650 reflections
226 parameters
H-atom parameters constrained
Δρ_max_ = 0.29 e Å^−3^
Δρ_min_ = −0.22 e Å^−3^

### 

Data collection: *SMART* (Bruker, 2002 ▶); cell refinement: *SAINT* (Bruker, 2002 ▶); data reduction: *SAINT*; program(s) used to solve structure: *SHELXS97* (Sheldrick, 2008 ▶); program(s) used to refine structure: *SHELXL97* (Sheldrick, 2008 ▶); molecular graphics: *SHELXTL* (Sheldrick, 2008 ▶); software used to prepare material for publication: *SHELXTL*.

## Supplementary Material

Crystal structure: contains datablocks global, I. DOI: 10.1107/S1600536809006084/bq2125sup1.cif

Structure factors: contains datablocks I. DOI: 10.1107/S1600536809006084/bq2125Isup2.hkl

Additional supplementary materials:  crystallographic information; 3D view; checkCIF report

## Figures and Tables

**Table:** 

Ni1—O1	1.8897 (12)
Ni1—O2	1.9125 (12)
Ni1—N1	1.9435 (15)
Ni1—N2	1.9507 (14)

**Table:** 

O1—Ni1—O2	89.22 (5)
O1—Ni1—N1	93.76 (5)
O2—Ni1—N1	175.27 (6)
O1—Ni1—N2	177.83 (6)
O2—Ni1—N2	92.71 (5)
N1—Ni1—N2	84.25 (6)

**Fig. 1.**
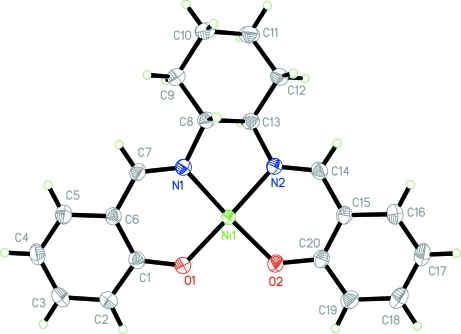
The structure of (I), showing the atom-numbering scheme. Displacement ellipsoids are drawn at the 30% probability level.

## supplementary crystallographic information

### Comment

Nickel(II) complexes play an important role in both bioinorganic chemistry and coordination chemistry (Suh *et al.*, 1996; Dey *et al.*, 2004; Angulo *et al.*, 2001; Ramadevi *et al.*, 2005; Edison *et al.*, 2004). As a further study of the structures of such complexes, the title mononuclear nickel(II) complex, (I), is reported in this paper.

In (I), the Ni atom is four-coordinated in a square planar geometry by the four donor atoms of the Schiff base ligand. The dihedral angle between the two benzene rings is 9.4 (2)°. The cyclohexyl group adopts C-form chair conformation with the generalized puckering coordinates; q(3)=-0.569 (1)Å, q(2)=0.009 (1)Å and φ=96.288 (1)° (Cremer & Pople, 1975) (Fig. 1).

### Experimental

Salicylaldehyde (0.2 mmol, 24.5 mg) and cyclohexyl-1,2-diamine (0.1 mmol, 11.4 mg) were dissolved in 10 ml methanol. To the mixture was added dropwise a 5 ml me thanol solution of nickel(II) nitrate hexahydrate (0.2 mmol, 58.2 mg) with stirring. The final solution was allowed to stand in air for two weeks, yielding red block-shaped crystals of (I).

### Refinement

H atoms were constrained to ideal geometries, with C—H = 0.93–0.97Å and *U*_iso_(H) = 1.2*U*_eq_(C).

### Crystal data

**Table:**

[Ni(C_20_H_20_N_2_O_2_)]	*F*(000) = 792
*M**_r_* = 379.09	*D*_x_ = 1.497 Mg m^−^^3^
Monoclinic, *P*2_1_/*c*	Mo *K*α radiation, λ = 0.71073 Å
*a* = 7.6193 (8) Å	Cell parameters from 3779 reflections
*b* = 19.118 (2) Å	θ = 2.6–28.6°
*c* = 11.5459 (12) Å	µ = 1.17 mm^−^^1^
β = 90.907 (1)°	*T* = 298 K
*V* = 1681.6 (3) Å^3^	Block, red
*Z* = 4	0.30 × 0.30 × 0.28 mm

### Data collection

**Table:**

Bruker SMART CCD area-detector diffractometer	3650 independent reflections
Radiation source: fine-focus sealed tube	3023 reflections with *I* > 2σ(*I*)
graphite	*R*_int_ = 0.022
ω scans	θ_max_ = 27.0°, θ_min_ = 2.1°
Absorption correction: multi-scan (*SADABS*; Sheldrick, 1996)	*h* = −9→9
*T*_min_ = 0.721, *T*_max_ = 0.735	*k* = −24→24
9694 measured reflections	*l* = −14→9

### Refinement

**Table:**

Refinement on *F*^2^	Primary atom site location: structure-invariant direct methods
Least-squares matrix: full	Secondary atom site location: difference Fourier map
*R*[*F*^2^ > 2σ(*F*^2^)] = 0.027	Hydrogen site location: inferred from neighbouring sites
*wR*(*F*^2^) = 0.069	H-atom parameters constrained
*S* = 1.05	*w* = 1/[σ^2^(*F*_o_^2^) + (0.033*P*)^2^ + 0.2023*P*] where *P* = (*F*_o_^2^ + 2*F*_c_^2^)/3
3650 reflections	(Δ/σ)_max_ = 0.001
226 parameters	Δρ_max_ = 0.28 e Å^−^^3^
0 restraints	Δρ_min_ = −0.22 e Å^−^^3^

### Special details

**Table:**

Geometry. All e.s.d.'s (except the e.s.d. in the dihedral angle between two l.s. planes) are estimated using the full covariance matrix. The cell e.s.d.'s are taken into account individually in the estimation of e.s.d.'s in distances, angles and torsion angles; correlations between e.s.d.'s in cell parameters are only used when they are defined by crystal symmetry. An approximate (isotropic) treatment of cell e.s.d.'s is used for estimating e.s.d.'s involving l.s. planes.
Refinement. Refinement of *F*^2^ against ALL reflections. The weighted *R*-factor *wR* and goodness of fit *S* are based on *F*^2^, conventional *R*-factors *R* are based on *F*, with *F* set to zero for negative *F*^2^. The threshold expression of *F*^2^ > σ(*F*^2^) is used only for calculating *R*-factors(gt) *etc*. and is not relevant to the choice of reflections for refinement. *R*-factors based on *F*^2^ are statistically about twice as large as those based on *F*, and *R*- factors based on ALL data will be even larger.

### Fractional atomic coordinates and isotropic or equivalent isotropic displacement parameters (Å^2^)

**Table:**

	*x*	*y*	*z*	*U*_iso_*/*U*_eq_	
Ni1	0.80232 (3)	0.468244 (10)	0.481224 (17)	0.03293 (8)	
O1	0.86946 (19)	0.37326 (6)	0.49143 (10)	0.0477 (3)	
O2	0.89546 (18)	0.48282 (6)	0.63411 (11)	0.0484 (3)	
N1	0.6899 (2)	0.45709 (7)	0.33007 (12)	0.0378 (3)	
N2	0.7259 (2)	0.56527 (7)	0.46659 (12)	0.0386 (3)	
C1	0.8626 (2)	0.32645 (9)	0.40841 (15)	0.0395 (4)	
C2	0.9388 (3)	0.26009 (9)	0.42918 (17)	0.0472 (5)	
H2	0.9899	0.2508	0.5012	0.057*	
C3	0.9392 (3)	0.20924 (9)	0.34579 (18)	0.0500 (5)	
H3	0.9903	0.1661	0.3625	0.060*	
C4	0.8651 (3)	0.22058 (10)	0.23682 (18)	0.0526 (5)	
H4	0.8671	0.1859	0.1804	0.063*	
C5	0.7887 (3)	0.28423 (9)	0.21422 (17)	0.0488 (5)	
H5	0.7383	0.2923	0.1415	0.059*	
C6	0.7842 (2)	0.33781 (9)	0.29806 (15)	0.0386 (4)	
C7	0.6995 (2)	0.40218 (9)	0.26570 (15)	0.0412 (4)	
H7	0.6473	0.4044	0.1924	0.049*	
C8	0.5917 (3)	0.52030 (8)	0.29253 (16)	0.0401 (4)	
H8	0.4767	0.5185	0.3292	0.048*	
C9	0.5592 (3)	0.52841 (9)	0.16326 (16)	0.0482 (5)	
H9A	0.4894	0.4895	0.1346	0.058*	
H9B	0.6703	0.5281	0.1233	0.058*	
C10	0.4630 (3)	0.59708 (10)	0.13887 (18)	0.0565 (5)	
H10A	0.4499	0.6032	0.0558	0.068*	
H10B	0.3464	0.5947	0.1712	0.068*	
C11	0.5588 (3)	0.65940 (10)	0.18949 (17)	0.0555 (5)	
H11A	0.4900	0.7013	0.1755	0.067*	
H11B	0.6703	0.6649	0.1512	0.067*	
C12	0.5911 (3)	0.65080 (9)	0.31981 (16)	0.0488 (5)	
H12A	0.6590	0.6901	0.3490	0.059*	
H12B	0.4797	0.6502	0.3594	0.059*	
C13	0.6899 (2)	0.58284 (9)	0.34426 (15)	0.0419 (4)	
H13	0.8032	0.5860	0.3056	0.050*	
C14	0.7157 (2)	0.60977 (9)	0.54952 (16)	0.0423 (4)	
H14	0.6733	0.6541	0.5315	0.051*	
C15	0.7650 (2)	0.59611 (9)	0.66825 (15)	0.0397 (4)	
C16	0.7300 (3)	0.64851 (10)	0.75078 (17)	0.0478 (5)	
H16	0.6726	0.6890	0.7268	0.057*	
C17	0.7777 (3)	0.64176 (10)	0.86443 (17)	0.0551 (5)	
H17	0.7512	0.6766	0.9175	0.066*	
C18	0.8670 (3)	0.58153 (11)	0.89957 (17)	0.0547 (5)	
H18	0.9010	0.5764	0.9768	0.066*	
C19	0.9054 (3)	0.52957 (10)	0.82153 (16)	0.0478 (5)	
H19	0.9663	0.4902	0.8469	0.057*	
C20	0.8544 (2)	0.53471 (8)	0.70409 (15)	0.0398 (4)	

### Atomic displacement parameters (Å^2^)

**Table:**

	*U*^11^	*U*^22^	*U*^33^	*U*^12^	*U*^13^	*U*^23^
Ni1	0.03599 (14)	0.03109 (12)	0.03155 (13)	0.00174 (9)	−0.00469 (9)	−0.00265 (8)
O1	0.0670 (9)	0.0363 (6)	0.0396 (7)	0.0052 (6)	−0.0079 (6)	−0.0018 (5)
O2	0.0549 (9)	0.0475 (7)	0.0424 (7)	0.0133 (6)	−0.0129 (6)	−0.0091 (6)
N1	0.0390 (9)	0.0359 (7)	0.0382 (8)	−0.0005 (6)	−0.0033 (6)	−0.0002 (6)
N2	0.0408 (9)	0.0366 (7)	0.0383 (8)	0.0000 (6)	−0.0041 (6)	−0.0020 (6)
C1	0.0409 (10)	0.0362 (9)	0.0415 (10)	−0.0038 (7)	0.0029 (8)	0.0004 (7)
C2	0.0541 (12)	0.0384 (9)	0.0491 (11)	0.0025 (8)	−0.0001 (9)	0.0025 (8)
C3	0.0547 (13)	0.0345 (9)	0.0609 (13)	0.0020 (8)	0.0081 (10)	0.0003 (8)
C4	0.0640 (14)	0.0391 (10)	0.0548 (13)	−0.0022 (9)	0.0059 (10)	−0.0113 (9)
C5	0.0580 (13)	0.0442 (10)	0.0442 (11)	−0.0040 (9)	−0.0020 (9)	−0.0057 (8)
C6	0.0406 (10)	0.0365 (9)	0.0388 (9)	−0.0046 (7)	0.0013 (8)	−0.0021 (7)
C7	0.0441 (11)	0.0425 (9)	0.0369 (10)	−0.0049 (8)	−0.0055 (8)	−0.0036 (8)
C8	0.0400 (10)	0.0393 (9)	0.0410 (10)	0.0001 (7)	−0.0040 (8)	0.0013 (7)
C9	0.0525 (12)	0.0489 (11)	0.0429 (11)	0.0013 (9)	−0.0084 (9)	−0.0008 (8)
C10	0.0652 (14)	0.0527 (11)	0.0511 (12)	0.0051 (10)	−0.0157 (10)	0.0055 (9)
C11	0.0613 (14)	0.0498 (11)	0.0550 (12)	0.0024 (10)	−0.0072 (10)	0.0114 (9)
C12	0.0562 (13)	0.0391 (10)	0.0507 (11)	0.0022 (9)	−0.0070 (9)	0.0029 (8)
C13	0.0437 (11)	0.0412 (9)	0.0408 (10)	−0.0031 (8)	−0.0016 (8)	0.0030 (7)
C14	0.0443 (11)	0.0349 (9)	0.0476 (11)	−0.0004 (8)	−0.0023 (8)	−0.0030 (8)
C15	0.0389 (10)	0.0396 (9)	0.0406 (10)	−0.0043 (8)	0.0003 (8)	−0.0051 (7)
C16	0.0510 (12)	0.0420 (10)	0.0506 (12)	−0.0018 (9)	0.0021 (9)	−0.0090 (8)
C17	0.0660 (14)	0.0536 (11)	0.0458 (12)	−0.0066 (10)	0.0056 (10)	−0.0159 (9)
C18	0.0616 (14)	0.0647 (13)	0.0377 (11)	−0.0109 (10)	−0.0017 (9)	−0.0072 (9)
C19	0.0515 (12)	0.0506 (11)	0.0413 (11)	−0.0033 (9)	−0.0053 (9)	−0.0010 (8)
C20	0.0376 (10)	0.0426 (9)	0.0391 (10)	−0.0050 (8)	−0.0010 (8)	−0.0047 (8)

### Geometric parameters (Å, °)

**Table:**

Ni1—O1	1.8897 (12)	C9—C10	1.528 (2)
Ni1—O2	1.9125 (12)	C9—H9A	0.9700
Ni1—N1	1.9435 (15)	C9—H9B	0.9700
Ni1—N2	1.9507 (14)	C10—C11	1.510 (3)
O1—C1	1.312 (2)	C10—H10A	0.9700
O2—C20	1.320 (2)	C10—H10B	0.9700
N1—C7	1.289 (2)	C11—C12	1.530 (3)
N1—C8	1.483 (2)	C11—H11A	0.9700
N2—C14	1.284 (2)	C11—H11B	0.9700
N2—C13	1.473 (2)	C12—C13	1.526 (2)
C1—C2	1.414 (2)	C12—H12A	0.9700
C1—C6	1.415 (2)	C12—H12B	0.9700
C2—C3	1.368 (2)	C13—H13	0.9800
C2—H2	0.9300	C14—C15	1.439 (2)
C3—C4	1.388 (3)	C14—H14	0.9300
C3—H3	0.9300	C15—C16	1.411 (2)
C4—C5	1.372 (3)	C15—C20	1.416 (2)
C4—H4	0.9300	C16—C17	1.362 (3)
C5—C6	1.410 (2)	C16—H16	0.9300
C5—H5	0.9300	C17—C18	1.394 (3)
C6—C7	1.437 (2)	C17—H17	0.9300
C7—H7	0.9300	C18—C19	1.376 (3)
C8—C9	1.517 (2)	C18—H18	0.9300
C8—C13	1.527 (2)	C19—C20	1.408 (3)
C8—H8	0.9800	C19—H19	0.9300
			
O1—Ni1—O2	89.22 (5)	H9A—C9—H9B	108.2
O1—Ni1—N1	93.76 (5)	C11—C10—C9	112.26 (17)
O2—Ni1—N1	175.27 (6)	C11—C10—H10A	109.2
O1—Ni1—N2	177.83 (6)	C9—C10—H10A	109.2
O2—Ni1—N2	92.71 (5)	C11—C10—H10B	109.2
N1—Ni1—N2	84.25 (6)	C9—C10—H10B	109.2
C1—O1—Ni1	127.04 (11)	H10A—C10—H10B	107.9
C20—O2—Ni1	125.90 (11)	C10—C11—C12	111.42 (16)
C7—N1—C8	121.87 (15)	C10—C11—H11A	109.3
C7—N1—Ni1	125.39 (12)	C12—C11—H11A	109.3
C8—N1—Ni1	112.73 (10)	C10—C11—H11B	109.3
C14—N2—C13	123.45 (15)	C12—C11—H11B	109.3
C14—N2—Ni1	125.94 (13)	H11A—C11—H11B	108.0
C13—N2—Ni1	110.53 (10)	C13—C12—C11	110.17 (16)
O1—C1—C2	118.46 (16)	C13—C12—H12A	109.6
O1—C1—C6	124.38 (16)	C11—C12—H12A	109.6
C2—C1—C6	117.16 (16)	C13—C12—H12B	109.6
C3—C2—C1	121.58 (18)	C11—C12—H12B	109.6
C3—C2—H2	119.2	H12A—C12—H12B	108.1
C1—C2—H2	119.2	N2—C13—C12	117.09 (15)
C2—C3—C4	121.41 (18)	N2—C13—C8	106.20 (13)
C2—C3—H3	119.3	C12—C13—C8	110.91 (15)
C4—C3—H3	119.3	N2—C13—H13	107.4
C5—C4—C3	118.45 (18)	C12—C13—H13	107.4
C5—C4—H4	120.8	C8—C13—H13	107.4
C3—C4—H4	120.8	N2—C14—C15	124.88 (16)
C4—C5—C6	121.89 (19)	N2—C14—H14	117.6
C4—C5—H5	119.1	C15—C14—H14	117.6
C6—C5—H5	119.1	C16—C15—C20	119.10 (16)
C5—C6—C1	119.49 (16)	C16—C15—C14	117.73 (16)
C5—C6—C7	117.35 (16)	C20—C15—C14	123.08 (15)
C1—C6—C7	123.16 (15)	C17—C16—C15	122.23 (18)
N1—C7—C6	125.24 (16)	C17—C16—H16	118.9
N1—C7—H7	117.4	C15—C16—H16	118.9
C6—C7—H7	117.4	C16—C17—C18	118.67 (18)
N1—C8—C9	116.41 (14)	C16—C17—H17	120.7
N1—C8—C13	106.37 (14)	C18—C17—H17	120.7
C9—C8—C13	112.08 (15)	C19—C18—C17	120.90 (19)
N1—C8—H8	107.2	C19—C18—H18	119.5
C9—C8—H8	107.2	C17—C18—H18	119.5
C13—C8—H8	107.2	C18—C19—C20	121.48 (18)
C8—C9—C10	109.85 (15)	C18—C19—H19	119.3
C8—C9—H9A	109.7	C20—C19—H19	119.3
C10—C9—H9A	109.7	O2—C20—C19	118.21 (16)
C8—C9—H9B	109.7	O2—C20—C15	124.17 (16)
C10—C9—H9B	109.7	C19—C20—C15	117.61 (16)
